# Non-invasive respiratory volume monitoring for quantification of respiratory depression after benzodiazepine administration

**DOI:** 10.1186/cc14308

**Published:** 2015-03-16

**Authors:** G Mullen, D Ladd

**Affiliations:** 1 Vidant Medical Center, Greenville, NC, USA; 2 Respiratory Motion, Inc., Waltham, MA, USA

## Introduction

Benzodiazepines are used in many of settings to induce sedation, but can cause a reduction in respiratory drive. Objective monitoring of the effect of benzodiazepines on respiratory status in non-intubated patients has been difficult, putting patient safety at risk. A non-invasive respiratory volume monitor (RVM) that provides continuous measurement of minute ventilation (MV), tidal volume (TV) and respiratory rate (RR) was used to quantify the effects of midazolam on respiratory status in spontaneously breathing patients.

## Methods

An impedance-based RVM (ExSpiron; Respiratory Motion Inc., Waltham, MA, USA) was used in 30 patients who received 2 mg midazolam prior to induction of anesthesia and were sedated but spontaneously breathing. Eleven of these patients (58 ± 19 years, average BMI 27.7) received midazolam at least 20 minutes prior to induction. Digital RVM data were collected and MV, TV and RR calculated and evaluated from 30-second segments 10 minutes before and after the first dose of midazolam. Ten patients were analyzed as a group and one patient was analyzed separately (due to idiosyncratic reaction).

## Results

**Figure 1:**
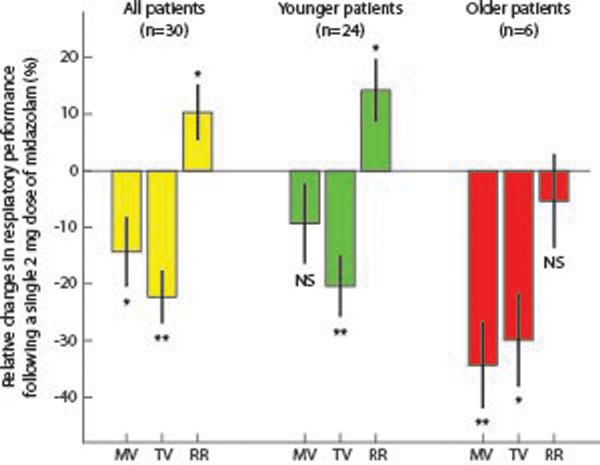


Following administration of midazolam, the group MV and TV decreased an average of 19 ± 7% and 16 ± 5%, respectively (mean ± SEM, *P *< 0.01, both) while RR remained essentially unchanged (decrease of 3 ± 8%, *P >*0.3). In the younger half of the cohort (45 ± 16 years), the decreases in MV and TV were not significant, only 6 ± 3% and 8 ± 5%, respectively. The older half of the cohort (72 ± 8 years) displayed fourfold greater MV and TV decreases (32 ± 11%, *P *< 0.05 and 25 ± 6%, *P *< 0.05), when compared with the younger cohort, *P *< 0.01, Figure 1).

## Conclusion

Continuous monitoring with RVM provides a valuable depiction of hypoventilation from benzodiazepines, not demonstrated by other methodologies such as pulse oximetry and RR alone. RVM monitoring can help uncover potentially life-threatening hypoventilation in older patients. Further studies are ongoing to quantify hypoventilation after administration of other anesthetic medications.

